# Supplementary figures and images for: The multi metal-resistant bacterium Cupriavidus metallidurans CH34 affects growth and metal mobilization in Arabidopsis thaliana plants exposed to copper
Source: PeerJ. 2021 May 14;9:e11373. doi: 10.7717/peerj.11373 (PMC8127957; doi:10.7717/peerj.11373)

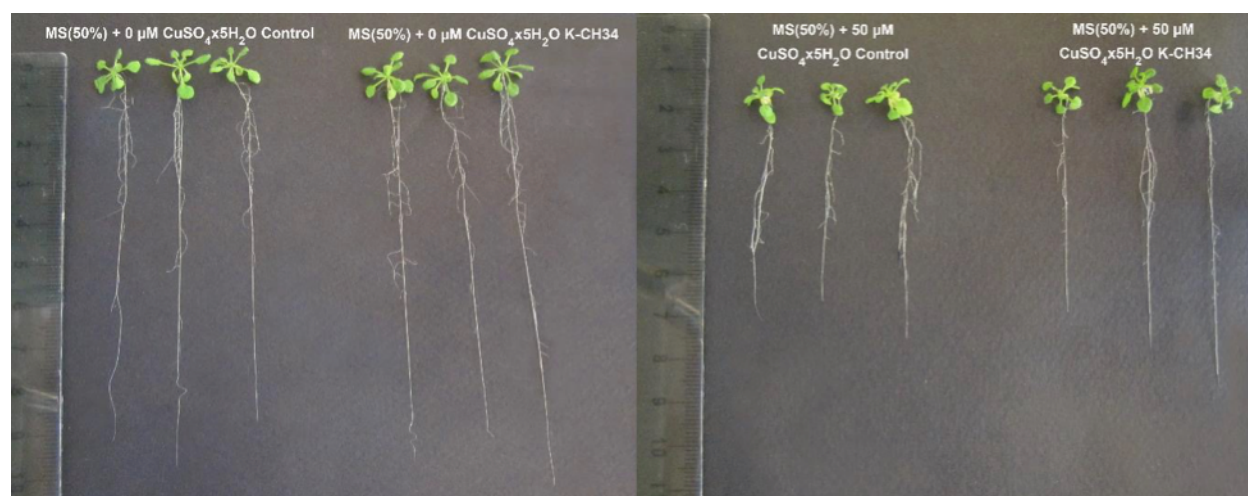

Supplement: Supplemental Information 1 — Representative 21 days after sowing A. thaliana individuals, inoculated or not with heat-inactivated cells of C. metallidurans (K-CH34) in the absence (left side), or the presence (right side) of copper (50 µM CuSO4x5H2O). MS: Murashige—Skoog plant growth medium. [file peerj-09-11373-s001.pdf]

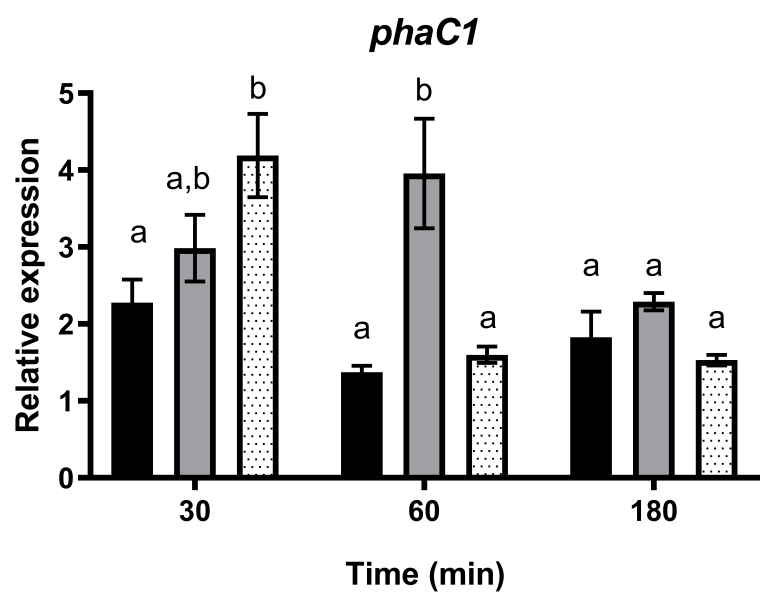

■ Plant + 25  $\mu\text{M}$   $\text{Cu}^{+2}$     ■ Plant (At)    ■ 25  $\mu\text{M}$   $\text{Cu}^{+2}$

Supplement: Supplemental Information 2 — Quantitative Real Time Polymerase Chain Reactions determinations of relative expression levels were normalized respect to the housekeeping gene 16S rRNA. Data correspond to means ± standard deviations of at least three biological replicates. Different letters indicate significant differences between same time conditions (Two way ANOVA Tukey’s HSD tests; p < 0.05). [file peerj-09-11373-s002.pdf]
