# Supplementary material for: The multi metal-resistant bacterium Cupriavidus metallidurans CH34 affects growth and metal mobilization in Arabidopsis thaliana plants exposed to copper
Source: PeerJ. 2021 May 14;9:e11373. doi: 10.7717/peerj.11373 (PMC8127957; doi:10.7717/peerj.11373)
Supplement: Supplemental Information 3 [file peerj-09-11373-s003.docx]

**Supplementary Material**

**The multi metal-resistant bacterium *Cupriavidus metallidurans* CH34 affects growth and metal mobilization in *Arabidopsis thaliana* plants exposed to copper**

Claudia Clavero-León^1,2^, Daniela Ruiz^1,2^, Javier Cillero^1,2^, Julieta Orlando^3^, and Bernardo González^1,2^

FIGURE LEGENDS

**Supplementary Figure S1:** **Effects of inoculation of *Arabidopsis thaliana* with heat-inactivated *Cupriavidus metallidurans* cells, in the presence or in the absence of copper.** Representative 21 days after sowing *A. thaliana* individuals, inoculated or not with heat-inactivated cells of *C. metallidurans* (K-CH34) in the absence (left side), or the presence (right side) of copper (50 μM CuSO4x5H2O). MS: Murashige - Skoog plant growth medium

**Supplementary Figure S2:** **Relative expression levels of *phaC1* gene from *Cupriavidus metallidurans* CH34, in the presence or in the absence of *Arabidopsis thaliana* or copper.** Quantitative Real Time Polymerase Chain Reactions determinations of relative expression levels were normalized respect to the housekeeping gene 16S rRNA. Data correspond to means ± standard deviations of at least three biological replicates. Different letters indicate significant differences between same time conditions (Two way ANOVA Tukey's HSD tests; p < 0.05).

MANUSCRIPT FILE

Copy for inspection of a manuscript (Cillero *et al*.) under revision, supporting evidence that strain CH34 is able to grow on *Arabidopsis thaliana* roots exudates.
